# Diagnostic accuracy of the core components of a multi-parametric cardiovascular magnetic resonance imaging protocol: a CE-MARC sub-study

**DOI:** 10.1186/1532-429X-16-S1-O19

**Published:** 2014-01-16

**Authors:** John P Greenwood, David P Ripley, Manish Motwani, Julia Brown, Jane Nixon, Colin C Everett, Neil Maredia, Petra Bijsterveld, Sven Plein

**Affiliations:** 1 Multidisciplinary Cardiovascular Research Centre (MCRC) & Leeds Institute of Genetics, Health and Therapeutics, University of Leeds, Leeds, UK; 2 Clinical Trials Research Unit, University of Leeds, Leeds, UK

## Background

The CE-MARC study was the largest prospective evaluation of the diagnostic accuracy of cardiovascular magnetic resonance (CMR) in coronary artery disease (CAD), demonstrating its superiority over single-photon emission computed tomography [1]. The trial adopted a multi-parametric protocol assessing ventricular function, myocardial perfusion, viability (with late gadolinium enhancement (LGE)) and coronary artery anatomy. There have been a number of previous studies analysing the diagnostic accuracy of different components of the CMR examination with contrasting results. We assessed the diagnostic accuracy of the individual components and selected combinations of the multi-parametric CMR examination from the CE-MARC study.

## Methods

All patients from the CE-MARC population were studied. Visual CMR analyses were from the original, blinded read. Pre-specified sub-analysis of the four individual core components of the CMR protocol was performed in isolation, as a paired component (perfusion and LGE) and as a triplet (perfusion, LGE and ventricular function) and compared to the full multi-parametric protocol.

## Results

**Figure 1:**
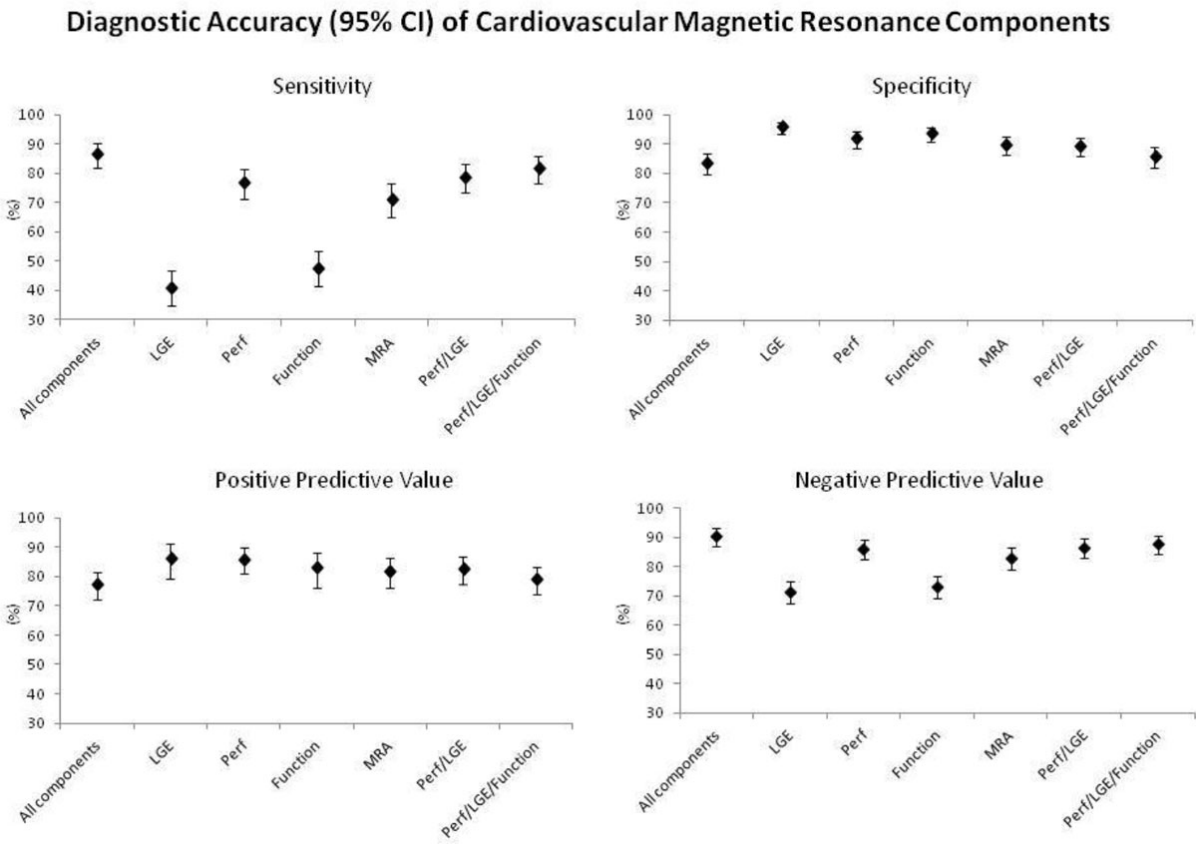
**Diagnostic accuracy of the combinations of Cardiovascular Magnetic Resonance Components in the CE-MARC study**. LGE - late gadolinium enhancement; Perf - perfusion; MRA - magnetic resonance angiography;

Both CMR and X-ray angiography were available in 676 patients. The sensitivity of the combined CMR protocol was 86.5%, specificity 83.4%, PPV 77.2% and NPV 90.5%. The diagnostic accuracy of the individual components and paired and triplet combinations compared to the full multi-parametric protocol are presented in Table 1 and Figure 1. The maximum sensitivity for the detection of significant CAD by CMR was achieved when all four components were used. No individual component; paired perfusion with LGE; or perfusion with LGE and function significantly outperformed the multi-parametric protocol in terms of sensitivity. However in terms specificity, the individual components of perfusion, ventricular function and late gadolinium enhancement (LGE) all performed significantly better than the multi-parametric protocol (P < 0.0001). In addition, combining LGE with perfusion or with perfusion and ventricular function significantly improved the test specificity compared to the multi-parametric protocol (P < 0.0001 for each). In terms of PPV and NPV, the multi-parametric protocol performed significantly better than all individual components, paired or triplet combination.

**Table 1:** Diagnostic accuracy of a multi-parametric CMR exam and its core components compared to the reference test X-ray angiography.

	Sensitivity (95%CI)	Specificity (95%CI)	PPV (95%CI)	NPV (95%CI)
Overall multi-parametric CMR study (all components) (n = 676)	86.5 (81.8, 90.1)	83.4 (79.5, 86.7)	77.2 (72.1, 81.6)	90.5 (87.1, 93.0)

**Single CMR components**				

LGE (n = 674)	40.8 (35.0, 46.8)	95.8 (93.4, 97.4)	86.4 (79.3, 91.3)	71.4 (67.5, 75.0)
Perfusion (n = 661)	76.9 (71.4, 81.6)	91.8 (88.7, 94.1)	85.8 (80.8, 89.7)	86.0 (82.4, 89.0)
Ventricular function (n = 676)	47.4 (41.4, 53.4)	93.7 (90.9, 95.6)	82.9 (76.1, 88.1)	73.3 (69.3, 76.9)
MRA (n = 597) **	71.2 (65.1, 76.7)	89.8 (86.3, 92.5)	81.8 (75.9, 86.5)	83.0 (79.0, 86.4)

**Combinations**				

Perfusion/LGE (n = 676)	78.6 (73.3, 83.1)	89.3 (85.9, 91.9)	82.6 (77.5, 86.8)	86.5 (82.9, 89.5)
Perfusion/LGE/ventricular function (n = 676)	81.6 (76.5, 85.8)	85.9 (82.1, 88.9)	78.9 (73.7, 83.3)	87.8 (84.2, 90.6)

CMR - cardiovascular magnetic resonance; LGE - late gadolinium enhancement; MRA - magnetic resonance angiography. ** Only includes excellent or adequate quality MRA

## Conclusions

A combined multi-parametric CMR protocol has higher sensitivity, PPV and NPV that the individual components however the specificity of the individual components of perfusion, ventricular function and late gadolinium enhancement (LGE) all performed significantly better than the multi-parametric protocol.

## Funding

This study was funded by the British Heart Foundation (RG/05/004). J.P.G and S.P. received research grant from Philips Healthcare. S.P. is funded by a British Heart Foundation fellowship (FS/10/62/28409).
